# A novel factor graph-based optimization technique for stereo correspondence estimation

**DOI:** 10.1038/s41598-022-19336-9

**Published:** 2022-09-16

**Authors:** Hanieh Shabanian, Madhusudhanan Balasubramanian

**Affiliations:** Department of Electrical and Computer Engineering, The University of Memphis, Memphis, TN 38152 USA

**Keywords:** Applied mathematics, Computer science

## Abstract

Dense disparities among multiple views are essential for estimating the 3D architecture of a scene based on the geometrical relationship between the scene and the views or cameras. Scenes with larger extents of homogeneous textures, differing scene illumination among the multiple views and with occluding objects affect the accuracy of the estimated disparities. Markov random fields based methods for disparity estimation address these limitations using spatial dependencies among the observations and among the disparity estimates. These methods, however, are limited by spatially fixed and smaller neighborhood systems or cliques. Recent learning-based methods generate rich set of stereo features for generating cost volume and estimating disparity. In this work, we present a new factor graph-based probabilistic graphical model for disparity estimation that allows a larger and a spatially variable neighborhood structure determined based on the local scene characteristics. Our algorithm improves the accuracy of disparity estimates in stereo image pairs with varying texture and illumination characteristics by enforcing spatial dependencies among scene characteristics as well as among disparity estimates. We evaluated our method using the *Middlebury benchmark stereo datasets* and the *Middlebury evaluation dataset version 3.0* and compared its performance with recent state-of-the-art disparity estimation algorithms. Our factor graph-based algorithm provided disparity estimates with higher accuracy when compared to the recent non-learning- and learning-based disparity estimation algorithms. The factor graph formulation can be used for obtaining *maximum a posteriori* estimates from models or optimization problems with complex dependency structure among hidden variables. The strategies of using a priori distributions with shorter support and spatial dependencies were useful for reducing the computational cost and improving message convergence in the model. The factor-graph algorithm is also useful for other dense estimation problems such as optical flow estimation.

## Introduction

The 3D architecture of an object or a scene can be estimated from two or more views of the scene by determining dense correspondences among the multiple views of the scene. A disparity map comprised of dense pixel-level correspondences between images in a stereo pair can be estimated using stereo matching algorithms. Stereo disparity estimation has wide-ranging applications such as robot navigation^1^, aerial data analysis^2^, image sequence analysis^3^, and 3-D surface reconstruction^4^. The presence of large untextured regions (homogeneous intensity), occluding objects and uneven intensity distributions in the stereo pair of images introduce significant challenges in estimating stereo disparity maps.

Two broad categories of stereo matching methods are window-based and energy-based algorithms^5^. The energy-based algorithms^6^ are global methods with a cost function defined as a function of the entire image extent^7^. Window-based or local methods utilize a finite support window to define the cost function and are suitable for real-time applications. However, regions with homogeneous texture and occlusion affect the accuracy of the disparity estimated using local methods.

**Figure 1:**
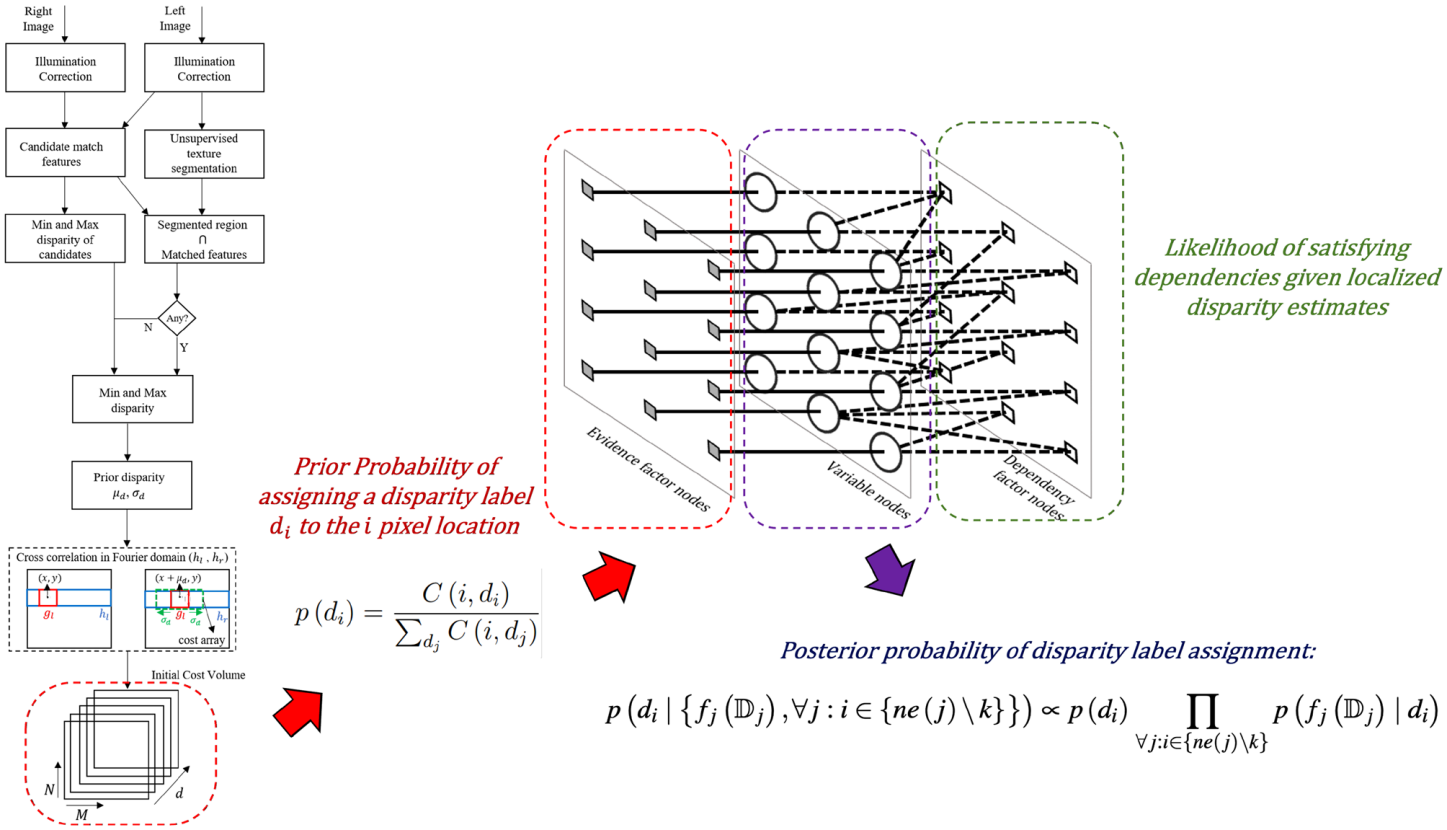
Overview of the FGS algorithm illustrating generation of a priori distribution from a stereo pair and calculating *a posterior* disparity distribution using our factor graph design. A detailed illustration of our factor graph architecture is shown in Fig. 2 and the sparse cost volume calculations used for building a priori disparity distributions are shown in Fig. 3.

In general, stereo disparity estimation procedures include an initial cost calculation step, a cost aggregation step, an optimal disparity estimation step, and a disparity refinement step. Some of the successful and commonly used disparity cost measures are normalized cross-correlation (NCC)^8^, sum of squared differences (SSD)^9^, sum of absolute differences (SAD)^10^, gradient-based measures^11^, feature-based measures^12^, rank transform (RT)^13^, and census transform (CT)^14^. Cost aggregation is useful for minimizing matching uncertainties and improving the accuracy of the estimates^15^. During cost aggregation, initial disparity cost measures within the support region of each pixel are aggregated^16^ such as using a low pass filter with a fixed kernel size or a variable support window (VSW)^17^, using adaptive support weight (ASW)^18^, and using a cross-based support window^19^. Using edge-preserving filters such as a bilateral filter (BF)^18,20^ and guided image filter (GIF)^21^ for cost aggregation provides a significant improvement on the final disparity estimates over the initial disparity estimates. An optimal disparity map or disparity estimates are obtained from the aggregated cost volume using optimization procedures such as the winner-take-all (WTA)^22^ or using global optimization procedures such as dynamic programming(DP)^23^, graph cut (GC)^24^, intrinsic curves^25^, and belief propagation^26^ methods.

Recently, learning-based methods have been successful in estimating stereo disparity with higher accuracy. Among the recent learning-based disparity estimation techniques, FC-DCNN is a light-weight, fully convolutional and densely connected neural network^27^ based on a densely connected convolution network architecture^28^ and a CNN based patch-matching technique for estimating stereo disparity^29^. FC-DCNN is a fully convolutional siamese neural network with individual network branches with shared weights for simultaneously processing left and right stereo images. With 5 convolutional layers and 64 filters in each layer, the network generated features were used to estimate similarity between a reference patch centered at each pixel in the left image and candidate patches from the right image in the stereo pair. For each pixel location, a cosine similarity measure was estimated using the corresponding feature vectors from the left and right channels of the network. The cost volume was filtered along all three dimensions and an initial disparity map was obtained using a winner-takes-all strategy. Accuracy of the initial disparity estimate at each pixel was assessed using a *left-right consistency check*. Inconsistent locations were identified and removed from the disparity map. Disparity values at these inconsistent locations were estimated based on classification of these locations as either part of homogeneous scene background or textured foreground objects, and using additional morphological processing of the disparity map. When compared to the MC-CNN-ACRT^29^, GC-Net^30^, and PSM-Net^31^, FC-DCNN is a lightweight network with 0.37 M trainable parameters with higher post-processing computational cost.

A successful strategy for improving accuracy of disparity estimates is to utilize spatial dependencies of the scene characteristics as well as of the disparity estimates. Among the probabilistic inference formulations for estimating dense geometric correspondences^32^, Markov Random Fields (MRF) based approaches have been successful in modeling spatial geometrical dependencies^33^. In brief, unknown true disparities among multiple views of a scene are modeled as random variables on the pixel lattice (random field) and dependence among random variables is assumed to follow Markov property. Thus, MRF represents a joint distribution of the random field using conditional distributions of each of the random variables. In learning-based MRF models, parameters of the MRF potential functions are either learned separately from training data^34,35^ or along with the unknown states of the random variables^36^.

One of the limitations of the MRF models is that the neighborhood system used for enforcing spatial dependencies needs to be *maximal*. Further, the chosen dependency structure is uniformly enforced for all the random variables. Therefore, pairwise cliques or $2 \times 2$ cliques are more commonly used in MRF models. While learning methods are available for optimizing MRF parameters and neighborhood structure, they are generally limited to specific tasks.

Establishing dense correspondences among geometrical coordinates of multiple views is an ill-posed problem due to scene occlusion. Occluded regions are locations that are visible only in one of the images in a stereo image pair. Therefore, it is not possible to directly estimate disparities in these locations. Other primary sources of errors in disparity estimation are presence of larger scene extents with smoother texture characteristics, differences in scene illumination with respect to the multiple views/observers, and presence of sharp depth or disparity boundaries.

In this paper, we present a new factor graph-based probabilistic graphical model (FGS algorithm) that provides more accurate estimates of stereo disparity by using spatial dependency of scene characteristics (image intensity) and of disparity estimates. Figure 1 shows a schematic representation of computational steps in the FGS algorithm for generating *a posterior* disparity estimates for each stereo image pair. From a reference (left) image in each stereo pair, locations of various scene elements (foreground objects, and background elements) were grouped into image segments. A sparse but highly confident set of disparity estimates within each segmented region were used to build a priori distribution of disparities within each segmented region. In a factor graph model, all pixel locations within an image segment were associated with the respective a priori disparity distribution. For each pixel location, *a posteriori* disparity was estimated by enforcing spatial dependency among disparity estimates using a larger but spatially variable neighborhood system. A larger neighborhood system improves disparity estimates in regions with smoother texture and occlusion. A spatially variable neighborhood system provides higher disparity contrast along the depth boundaries. A final disparity map was obtained by post-processing the *a posterior* disparity map generated by the factor graph.

The main contributions of this study are as follows:

- We present a new factor graph-based disparity estimation algorithm. To the best of our knowledge, this is the first application of factor graphs for estimating stereo and multi-view disparity.
- The probabilistic graphical model allows use of larger and spatially variable neighborhood structure for enforcing belief exchange and dependency among highly related neighboring pixel locations. While larger neighborhood systems are beneficial for improving disparity estimates, the computational cost of belief exchange among neighbors in a graphical model is prohibitively high. Our FGS framework allows use of larger neighborhood systems with reduced computational steps by retaining disparity-dependency relationship only among highly related neighboring locations.
- The FGS algorithm generates disparity maps with higher contrast along depth boundaries. This is possible because of our use of spatially-variable dependency structures while retaining dependency only with highly related neighboring locations (e.g. by discarding dependencies with pixels across an object or depth boundary).
- We present strategies for reducing computational cost and for accelerating convergence of belief message exchanges in the factor graph namely **1.** by reducing computational steps required for calculating belief measures in the factor graph by using binary potential functions; **2.** by reducing number of variables as well as their limits of summation for marginalizing joint distributions during belief propagation in the factor graph; and **3.** by generating a priori distributions with shorter support.
- The factor graph structure can be used for determining *a posteriori* estimates in optimization problems with complex dependency structure among hidden variables.

We demonstrate the performance of the proposed probabilistic factor graph model by conducting extensive experiments using the Middlebury benchmark stereo datasets^37–40^ and compare its performance with other state-of-the-art disparity estimation algorithms using Middlebury evaluation dataset version 3.0^5^.

The remainder of the paper is structured as follows. In Section "Probabilistic factor graph model for disparity estimation", we present a detailed description of the new probabilistic factor graph-based stereo disparity estimation (FGS) algorithm. We present our experimental results in Section "Experimental results and discussion" and conclude this work in Section "Conclusions".

## Probabilistic factor graph model for disparity estimation

For notational convenience, a linear index $i \in \left\{ 1, \ldots , MN \right\}$ was used to identity each of the pixels in images of size $M \times N$ pixels. Rectified stereo image pairs were corrected for any illumination differences using a homomorphic filter^41^. In brief, each image *I*(*i*) is modeled as an interaction between an illumination component *l*(*i*) and a reflectance component *r*(*i*) as $I(i) = l(i) \, r(i)$. Assuming that the illumination varies gradually over the imaging area, the illumination variation is subtracted from each image in the $\log$ domain using a high-pass filter. For each of the rectified and illumination corrected stereo image pairs, disparities between the left and right images were calculated by using the left image as reference.

**Table 1:** Table of symbols.

Symbol	Definition
$\mathbb {V}$	A set of variable nodes associated with disparity random field $\mathbb {D}$
$\mathbb {F}$	A set of all factor nodes
$\mathbb {E}$	A set of evidence factor nodes with a priori disparity distributions
$\mathbb {S}$	A set of dependency factor nodes to enforce spatial dependency
$d_i$	A random variable assigned to each variable node $i \in \mathbb {V}$
$\mathbb {D}$	A set of all disparity random variables
*ne*(*i*)	A set of neighboring nodes connected with any given node *i*
$ne(i)\setminus j$	A set of all neighboring nodes of *i* excluding node *j*
$\mathbb {D}_j = \left\{ d_i, \forall i \in \mathbb {V} : i \in ne\left( j \in \mathbb {F}\right) \right\}$	A collection of random variables associated with any factor node $j \in \mathbb {F}$
$\mathbb {D}_j\setminus i \subseteq \mathbb {D}$	A collection of random variables in $\mathbb {D}_j$ except $d_i$
$\psi _j, \, j \in \mathbb {E}$	An evidence potential function associated with factor node *j*
$\psi _k, \, k \in \mathbb {S}$	A dependency potential function associated with factor node *k*
*D*	Maximum number of disparity levels in a stereo pair
*Q*	Maximum number of variables nodes connected to a dependency factor node

### Graph structure and message passing for approximate inference

**Figure 2:**
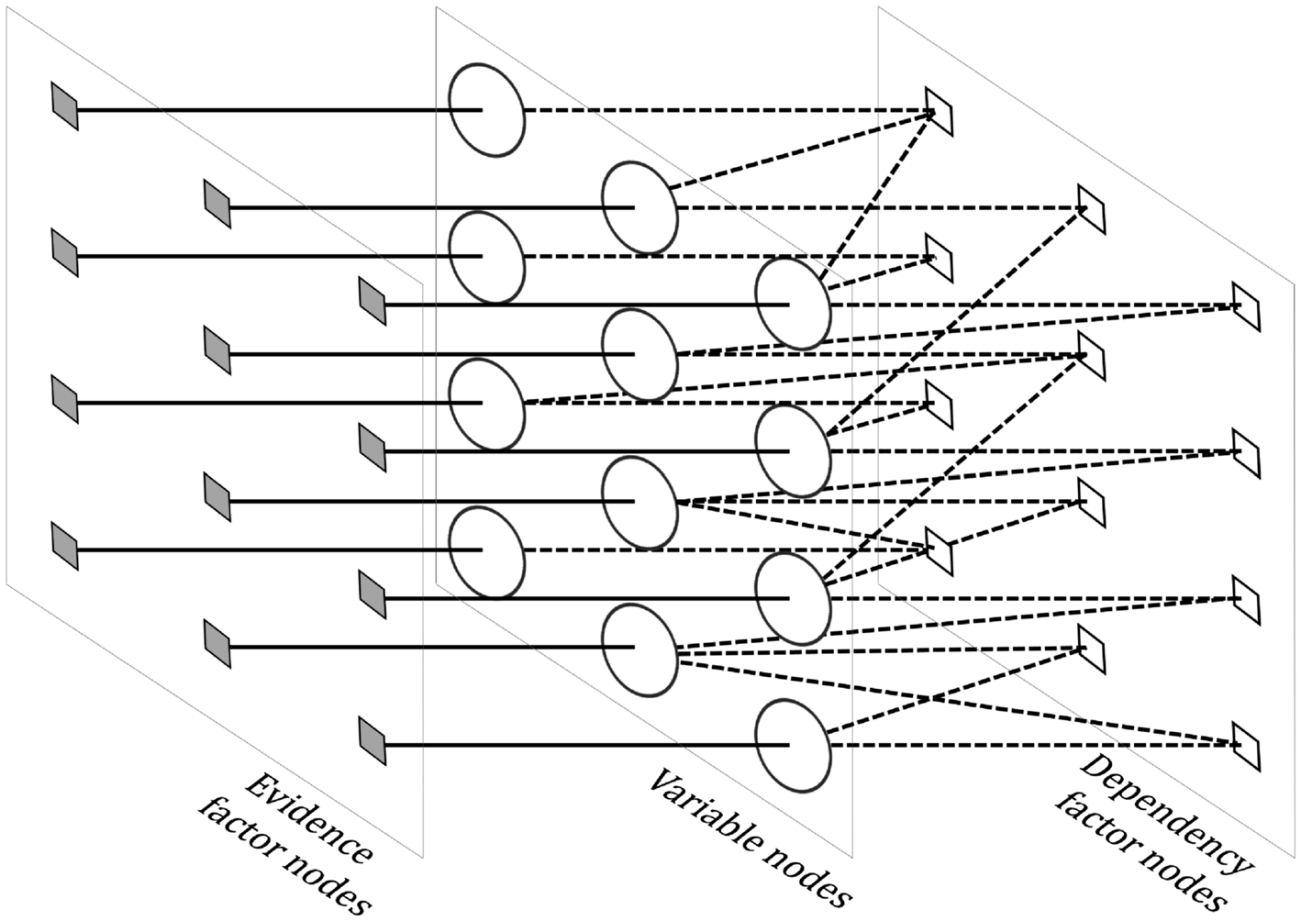
Schematic diagram of the proposed factor graph model (FGS algorithm) designed for optimal estimation of dense stereo disparities. For images of size $M \times N$, there are *MN* number of variable nodes (circular nodes) $\mathbb {V}$, *MN* number of dependency factor nodes (empty square nodes) $\mathbb {S}$ and *MN* number of evidence factor nodes (solid square nodes) $\mathbb {E}$. The variable nodes represent posterior disparities to be estimated; the evidence factor nodes represent prior information about the dense disparities; and the dependency factor nodes represent the likelihood of satisfying spatial dependencies given localized disparity estimates.

A list of symbols used in the remainder of the paper is presented in Table 1. Figure 2 shows the schematic diagram of a factor graph model designed for estimating a disparity map from each stereo image pair. A disparity map is comprised of disparities $\mathbb {D}= \left\{ d_i: d_i \in [d_{\min }, d_{\max } ] \subset \mathbb {Z} \right\} _{i=1}^{MN}$ at corresponding image pixel locations $\left\{ 1, \ldots , MN \right\}$ in the reference image. The bipartite graph is comprised of a set of variable nodes $\mathbb {V}$ and a set of factor nodes $\mathbb {F}= \mathbb {E}\cup \mathbb {S}$. The variable nodes $\mathbb {V}= \left\{ 1, \ldots , MN \right\}$ represent disparity labels assigned to each pixel. Evidence factor nodes $\mathbb {E}= \left\{ 1, \ldots , MN \right\}$ provide prior beliefs or evidences in assigning possible disparity labels to pixel locations. Dependency factor nodes $\mathbb {S}= \left\{ 1, \ldots , MN \right\}$ are used to model spatial dependencies among the disparity labels assigned to the neighboring pixels.

A random variable $d_i \in \mathbb {D}$ is assigned to each *variable node* $i \in \mathbb {V}$ to represent the disparity label assigned to the $i\hbox {th}$ pixel location. Each $j\hbox {th}$ *evidence factor node* in $\mathbb {E}$ is connected one-to-one with the corresponding $i\hbox {th}$ variable node in $\mathbb {V}$ to incorporate prior belief or evidence in determining the disparity labels $d_i$. To represent the influence of each pixel location on its neighboring pixels, each variable node $i \in \mathbb {V}$ is connected to one or more *dependency factor nodes* $\left\{ k \in \mathbb {S} \right\}$ using localized intensity characteristics in the reference image as described in Section "Determining variable nodes associated with each dependency factor node".

Let, *ne*(*i*) represent the set of neighboring nodes connected with any given node *i*; $ne(i)\setminus j$ represent a set of all neighboring nodes of *i* excluding node *j*, where $\setminus$ represents the set subtraction operation; $\mathbb {D}_j = \left\{ d_i, \forall i \in \mathbb {V}: i \in ne\left( j \in \mathbb {F}\right) \right\}$ be a collection of random variables associated with any factor node $j \in \mathbb {F}$; and let, $\mathbb {D}_j\setminus i \subseteq \mathbb {D}$ be a collection of random variables in $\mathbb {D}_j$ except $d_i$. An evidence potential function $\psi _j$ associated with each factor node $j \in \mathbb {E}$ is defined as a function of random variables $\mathbb {D}_j$ of its neighboring nodes $\psi _j\left( \mathbb {D}_j\right) = \psi _j\left( d_j\right)$. Similarly, the potential function $\psi _k(\mathbb {D}_k)$ at the $k\hbox {th}$ dependency factor node $k \in \mathbb {S}$ is defined as a function of the random variables $\mathbb {D}_k$ associated with its neighboring nodes. Therefore, the factor graph represents the joint distribution of disparity labels assigned to each of the pixel locations as

1 $$\begin{aligned} p\left( \mathbb {D}\right)&= \frac{1}{Z} \prod _{k \in \mathbb {F}} \psi _k\left( \mathbb {D}_k\right)&\nonumber \\&=\frac{1}{Z} \prod _{j \in \mathbb {E}} \psi _j\left( d_j\right) \prod _{k \in \mathbb {S}} \psi _k\left( \mathbb {D}_k\right)&\end{aligned}$$

where, *Z* is the partitioning function. Probability of assigning various disparity labels to each pixel *i* can be obtained by marginalizing Eq.  (1) with respect to $\mathbb {D}\setminus i$.

2 $$\begin{aligned} p\left( d_i\right) = \frac{1}{Z} \sum _{\mathbb {D}\setminus d_i} \quad \prod _{j \in \mathbb {E}} \psi _j\left( d_j\right) \prod _{k \in S} \psi _k\left( \mathbb {D}_k\right) \end{aligned}$$

This provides a sum-product formulation^42,43^ for determining likely disparity labels for each of the pixel locations *i* based on a priori disparity information and spatial dependency characteristics of disparities in a stereo image pair.

For an approximate and efficient computation of marginal beliefs or probabilities in Eq.  (2) using *loopy belief propagation*, local information available in each node is shared with neighboring nodes as variable-to-dependency factor messages $\mu _{i\in \mathbb {V}\rightarrow f\in \mathbb {S}}$ and factor-to-variable messages $\mu _{f\in \mathbb {F}\rightarrow i\in \mathbb {V}}$ until convergence^44,45^. Each outgoing message from a node is defined as a function of incoming messages at the given node as follows^44^.

3 $$\begin{aligned} \mu _{i\in \mathbb {V}\rightarrow f\in \mathbb {S}}&= \prod _{g \in \left\{ ne\left( i\right) \setminus f \right\} } \, \mu _{g \rightarrow i}\left( d_i\right)&\end{aligned}$$

4 $$\begin{aligned} \mu _{f\in \mathbb {F}\rightarrow i\in \mathbb {V}}&= \sum _{\mathbb {D}_f\setminus d_i} \, \psi _f\left( \mathbb {D}_f\right) \prod _{j \in \left\{ ne\left( f\right) \setminus i \right\} } \, \mu _{j\rightarrow f}\left( d_j\right)&\end{aligned}$$

### Probabilistic model

For approximate inference on disparity label assignment, message structures for loopy belief propagation in Eqs. (3) and (4) and relevant potential functions were defined based on the posterior probability of disparity label assignment,

$$\begin{aligned} p\left( d_i \mid \left\{ f_j\left( \mathbb {D}_j\right) , \forall j: i \in ne\left( j\right) \right\} \right) \propto p\left( \left\{ f_j\left( \mathbb {D}_j\right) , \forall j: i \in ne\left( j\right) \right\} \mid d_i\right) \, p\left( d_i\right) \end{aligned}$$

where, $f_j\left( \mathbb {D}_j\right)$ is a function of the state of all variable nodes neighboring the factor node *j*. In our proposed method, the most relevant and compact spatial dependencies are defined independently at each pixel *i* using local image characteristics as described in Section "Determining variable nodes associated with each dependency factor node". Therefore, the joint likelihood term can be simplified as

$$\begin{aligned} p\left( \left\{ f_j\left( \mathbb {D}_j\right) , \forall j: i \in ne\left( j\right) \right\} \mid d_i\right) = \prod _{\forall j: i \in ne\left( j\right) } p\left( f_j\left( \mathbb {D}_j\right) \mid d_i\right) \end{aligned}$$

and the posterior probability updated as

5 $$\begin{aligned} p\left( d_i \mid \left\{ f_j\left( \mathbb {D}_j\right) , \forall j: i \in ne\left( j\right) \right\} \right) \propto p\left( d_i\right) \, \prod _{\forall j: i \in ne\left( j\right) } p\left( f_j\left( \mathbb {D}_j\right) \mid d_i\right) \end{aligned}$$

It can be observed that the posterior probability of $d_i$ without conditioning on a dependency factor node $k \in ne\left( i\right)$

6 $$\begin{aligned} p\left( d_i \mid \left\{ f_j\left( \mathbb {D}_j\right) , \forall j: i \in \left\{ ne\left( j\right) \setminus k \right\} \right\} \right) \propto p\left( d_i\right) \, \prod _{\forall j: i \in \left\{ ne\left( j\right) \setminus k \right\} } p\left( f_j\left( \mathbb {D}_j\right) \mid d_i\right) \end{aligned}$$

resembles the structure of the variable-to-dependency factor node message $\mu _{i \rightarrow k}$ in Eq.  (3). This further suggests that individual likelihood terms $p\left( f_j\left( \mathbb {D}_j\right) \mid d_i\right)$ form the dependency factor node-to-variable node message structure in Eq.  (4).

Considering the individual likelihood terms in Eq.  (6),

$$\begin{aligned} p\left( f_j\left( \mathbb {D}_j\right) \mid d_i\right)&= \sum _{\mathbb {D}_j\setminus d_i} p\left( f_j\left( \mathbb {D}_j\right) , \mathbb {D}_j\setminus d_i \mid d_i\right)&\\&= \sum _{\mathbb {D}_j\setminus d_i} p\left( f_j\left( \mathbb {D}_j\right) \mid \mathbb {D}_j\right) \, p\left( \mathbb {D}_j\setminus d_i \mid d_i\right)&\\&= \sum _{\mathbb {D}_j\setminus d_i} p\left( f_j\left( \mathbb {D}_j\right) \right) \, p\left( \mathbb {D}_j\setminus d_i \mid d_i\right)&\end{aligned}$$

and assuming that random variables $\mathbb {D}_j$ associated with a dependency factor node $j \in \mathbb {S}$ are independent as in Moon & Gunther^46^,

7 $$\begin{aligned} p\left( f_j\left( \mathbb {D}_j\right) \mid d_i\right)&= \sum _{\mathbb {D}_j\setminus d_i} p\left( f_j\left( \mathbb {D}_j\right) \right) \, \prod _{d_k \in \mathbb {D}_j\setminus d_i} \, p\left( d_k \mid d_i\right)&\end{aligned}$$

In the absence of any loops between any two variable nodes *k* and *i* (i.e. when there is at most one common dependency factor node $s \in \mathbb {S}$ between any two variable nodes *k* and *i*), the conditional probability $p\left( d_k \mid d_i\right)$ can be interpreted as the posterior probability of $d_k$ conditioned on all dependency factor functions associated with the $k\hbox {th}$ variable node, i.e. $p\left( d_k \mid d_i\right) \propto p\left( d_k \mid \left\{ f_l\left( D_l\right) , \forall l : l \in ne\left( k\right) \right\} \right)$. By excluding the factor function $f_s\left( \mathbb {D}_s\right)$ associated with the factor node $s \in \left\{ ne\left( k\right) \cap ne\left( i\right) \right\}$ in $p\left( d_k \mid d_i\right)$, the individual likelihood expression in Eq.  (7) resembles the dependency factor node-to-variable node message structure $\mu _{j\rightarrow i}$ in Eq.  (4).

### Message passing implementation

Based on the message-passing structures in our factor graph model, the variable-to-dependency factor node message $\mu _{i\in \mathbb {V}\rightarrow f\in \mathbb {S}}$ in Eq.  (3) was approximated as the posterior probability of $d_i$ conditioned on (satisfying) all of its neighboring factor nodes except the factor node *f* to which the message is sent as in Eq. (6). Similarly, the factor node-to-variable node messages $\mu _{f\in \mathbb {F}\rightarrow i\in \mathbb {V}}$ in Eq.  (4) was approximated as the likelihood of satisfying spatial dependency among the random variable states of all variables nodes associated with the factor node *f* except $d_i$.

It can be observed that, for evidence factor nodes $f \in \mathbb {E}$, the factor-to-variable messages in Eq.  (4) simplifies as $\mu _{{f \in \mathbb {E}} \rightarrow i\in \mathbb {V}} = \psi _f\left( d_i\right)$. This supplies fixed prior information about the state $d_i$ of the *i*th variable node by restricting messages from variable nodes to only dependency factor nodes as in Eq.  (3). We estimated the a priori distribution $p\left( d_i\right)$ from a *disparity cost volume* containing the cost of assigning all possible disparity labels at each pixel location. Details of building the disparity cost volume is presented in Section 2.4. Therefore, the approximate inference obtained using our factor graph model provides updated disparities $\mathbb {D}$ (an optimal surface within the cost volume) based on their posterior probabilities.

The potential function $\psi _f\left( \mathbb {D}_f\right)$ in Eq. (4) and its probabilistic representation $p\left( f_j\left( \mathbb {D}_j\right) \right)$ in Eq. (7) at the spatial dependency factor node *j* was assigned a value of 1.0 when the states $\mathbb {D}_j \setminus i$ of the neighboring nodes are same as that of $d_i$; and was assigned a value of 0.0 when the states $\mathbb {D}_j \setminus i$ are different from that of $d_i$. This enforces spatial dependencies among the neighboring pixels in the final disparity map $\mathbb {D}$. In addition, this further reduces the number of marginalization operations in Eqs.  (4) and (7).

For each stereo image pairs, each evidence factor nodes $e \in \mathbb {E}$ were initialized with a priori probabilities $p\left( d_i\right)$ of the variable node $i \in ne\left( e\right)$ as the evidence factor node-to-variable node messages. The initial message from other variable and dependency factor nodes were set to be a uniform probability vector representing equally likely states. After initial message passing from the evidence factor nodes $\mathbb {E}$, message exchange continues among all graph nodes $\mathbb {V}\cup \mathbb {F}$ until message convergence. We utilized an $L_2$ measure of

8 $$\begin{aligned} \varepsilon ^{t+1} = \Vert \mathbb {D}^{t+1} - \mathbb {D}^t\Vert \end{aligned}$$

for assessing message convergence at message passing iteration $t+1$.

### Disparity cost volume and a priori disparity distribution $p\left( d_i\right)$

**Figure 3:**
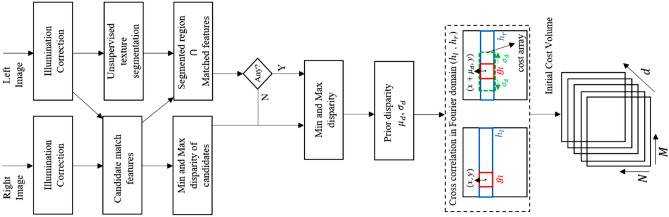
Schematic representation of sparse cost volume calculations.

For any given stereo image pair, let, $C\left( i, d_i\right)$ be a cost volume representing the cost of assigning a disparity label $d_i$ to location *i*. Thus the final disparity map for a given stereo image pair will be an optimal surface within the cost volume $C\left( i, d_i\right)$. Figure 3 shows a schematic representation of algorithmic steps used for disparity cost volume calculation.

For computationally efficient disparity estimation, the reference image was segmented using an unsupervised texture segmentation method^47^. In brief, sharp image segment boundaries were derived using a Gabor filter bank and image boundaries were aggregated using *k*-means clustering to generate an image segmentation map. Within each of the segmented regions, highly confident disparity estimates at several candidate locations were obtained using an eigen-based feature matching method^48^. Using the zonal/regional disparity distributions, disparity cost at each of the location *i* was calculated using a normalized cross-correlation measure in the frequency domain. Within each segmented region, only disparity labels ranging from $\left[ \mu _d - \sigma _d, \mu _d + \sigma _d\right]$ were considered based on the distribution of the disparity labels within the segmented region as shown in Fig. 3. This results in a sparse disparity cost volume $C(i, d_i)$ and thus facilitates a faster inference due to reduced marginalization limits in Eq. (2). A detailed description of the initial cost volume computation as part of a hybrid of cross-correlation and scene segmentation (HCS) algorithm is available elsewhere^49^.

A priori probability of assigning a disparity label $d_i$ to the $i\hbox {th}$ pixel location in the reference image was estimated from the cost volume $C\left( i, d_i\right)$ as

9 $$\begin{aligned} p\left( d_i\right) =\frac{C\left( i, d_i\right) }{\sum _{d_j} C\left( i, d_j\right) } \end{aligned}$$

### Determining variable nodes associated with each dependency factor node

We utilized edge-preserving filter kernels, namely the guided image filters (GIF)^50^ and bilateral filters (BF)^51^ to determine neighboring variable nodes with the highest influence on the true state of disparity at each of the $i\hbox {th}$ pixel locations. Neighborhood dependency information from these kernels were used to define a non-symmetric, irregular, and higher-order neighborhood system based on the fact that objects at various depths in the scene may exhibit a disparity boundary along the object boundary. Let $\left( x, y\right)$ represent the 2D coordinates of the $i\hbox {th}$ pixel. Highly dependent neighbors of location (*x*, *y*) were selected using an $\alpha \hbox {th}$ percentile cut-off of the kernel coefficients. Coordinates of these highly dependent neighbors were used to identify the neighboring variable nodes of each dependency factor node.

In brief, guided image filtering (GIF) is an edge-preserving smoothing algorithm. At each pixel location (*x*, *y*) in the reference image $\hat{r_l}$, a guided filter kernel $W_{xy}\left( i, j\right)$ with smoothness parameter $\epsilon$ was estimated as

10 $$\begin{aligned} W_{xy}\left( m, n\right) = \frac{1}{|\omega _{xy} |^2} \sum _{\forall \left( k, l\right) \in \omega _{xy}} \bigg (1 + \frac{(\hat{r_l}\left( m, n\right) - \mu _{xy})\left( \hat{r_l}\left( k,l\right) - \mu _{xy}\right) }{\sigma ^2_{xy} + \varepsilon } \bigg ) \end{aligned}$$

where $\omega _{xy}$ is the window size used for estimating local illumination characteristics namely the mean illumination $\mu _{xy}$ and standard deviation $\sigma$ at location $\left( x, y\right)$.

The Bilateral filter (BF)^51^ is an edge-preserving non-linear Gaussian filter with coefficients defined as a function of spatial and intensity similarities estimated respectively using a localized domain kernel and a range kernel. Bilateral filter kernel coefficients at location $\left( x, y\right)$ is given as

11 $$\begin{aligned} W_{xy}\left( m, n\right) = \exp \bigg (-\frac{\left( x - m\right) ^2 + \left( y - n\right) ^2}{2 \sigma ^2_d} - \frac{\Vert \hat{r_l}\left( x, y\right) - \hat{r_l}\left( m, n\right) \Vert ^2}{2\sigma ^2_r} \bigg ) \end{aligned}$$

where the first exponential term represents a domain kernel as a function of pixel distance with respect to the center pixel $\left( x, y\right)$ and the second term represents a range kernel as a function of regional image intensity with respect to that of the center pixel $\left( x, y\right)$. Parameters $\sigma _d$ and $\sigma _r$ controls the extent of influence neighboring pixels have on the domain and range filters respectively.

### Disparity estimates

Upon message passing convergence, an approximate estimate of the posterior probability of assigning a disparity label $d_i$ is available in each variable node as given in Eq.  (5). A *maximum a posterior* disparity estimate was determined at each pixel location *i* based on the disparity label $d_i$ with the maximum posterior probability at the respective pixel *i*.

### Post-processing the disparity maps

Occluded pixels were identified based on a lack of consistency or agreement between the disparity maps estimated using the left-right order vs right-left order of each stereo pair^52^. For each occluded pixel, the disparity estimate from its nearest non-occluded pixel within the same scanline (row) was assigned. Further, a weighted median filter was used to minimize spurious disparity assignments in the occluded region^53^.
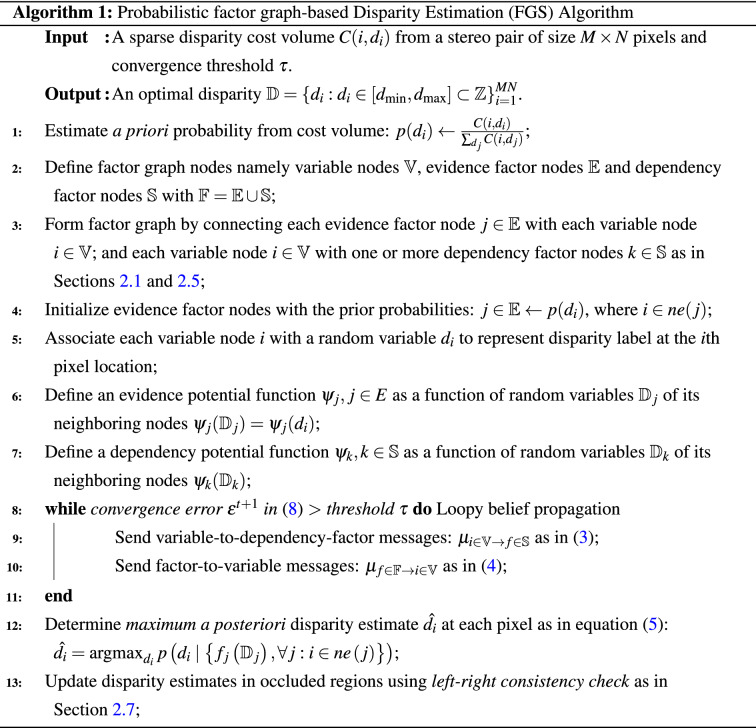


## Experimental results and discussion

Algorithmic steps of the proposed factor graph method are presented in Algorithm 1. We evaluated the proposed method using stereo images from the Middlebury benchmark stereo datasets^37–39^, and^40^. We present a detailed evaluation of the proposed FGS algorithm followed by a comprehensive comparison of its performance with other state-of-the-art disparity estimation algorithms using Middlebury evaluation dataset version 3.0^5^.

### FGS parameters and FGS implementation

The FGS algorithm was implemented in MATLAB 2018b and its performance was evaluated using an Intel(R) Xeon(R) workstation with E3-1271 v3, 3.6 GHz processor.

For illumination correction, high pass filters of size $21 \times 21$ pixels were obtained from a low-pass averaging filter. For unsupervised texture segmentation of the reference image, Gabor filters were designed^47^ with filter orientations between $0^{\circ }-150^{\circ }$ degrees in steps of $30^{\circ }$, and wavelength starting from 2.83 up to the magnitude of hypotenuse of the input image. These filter parameters were previously identified to provide localized frequency features (roughly orthogonal subset) and spatial orientation features from input images^47^. *K*-means clustering algorithm was initialized with $K=15$ with 5 replicates and ran for a maximum of 500 iterations. Based on our experiments, we observed that a higher number of clusters *K* are beneficial in reducing the computational complexity and for faster convergence of the FGS algorithm when there are several foreground objects at multiple depths in the scene. This is because of the fact that disparity values of locations within each distinct scene element are likely to be similar. Therefore, more accurate grouping of these scence elements will likely provide a priori disparity distributions with smaller standard deviations and shorter support leading to faster convergence of the FGS algorithm. While computational cost may be higher with non-optimal choice of clustering parameters the accuracy of the FGS disparity estimates is not affected.

We evaluated various image features to identify robust features necessary for building a priori disparity distributions. Among the minimum eigenvalue algorithm, speeded up robust features algorithm (SURF), Harris corner and edge detector algorithm, and accelerated segment test algorithm (FAST), we observed that the eigen-based feature matching method^48^ performed well with various types of benchmark images. For cost volume calculations, an optimal template size of $3 \times 3$ pixels were used^49^. For identifying variable nodes connected to each dependency factor node, bilateral filter kernel of size $7 \times 7$ pixels, domain kernel parameter of $\sigma _d = 3$, range kernel parameter of $\sigma _r = 0.1$ and coefficient percentile cut-off of $\alpha =97$ were used. We observed that the smallest kernel sizes along with a higher percentile cut-off $\alpha$ identified fewer but highly dependent neighboring nodes. Therefore, the computational cost of message passing was significantly reduced with fewer but highly reliable neighboring variable nodes connected to each dependency factor node.

### Computational complexity

In the FGS algorithm, message exchanges between the variable nodes $$\mathbb {V}$$ and the spatial dependency factor nodes $$\mathbb {S}$$ with $$|\mathbb {V} | = |\mathbb {S} | = MN$$ are computationally demanding. Let, *D* be the cardinality of sample space of the random variable $$d_i$$ associated with the *i*th variable node i.e. maximum number of disparity levels in a stereo pair. Let, *Q* be the maximum number of variable nodes connected to a dependency factor node. Preparing a message for sending from a factor node *f* to a variable node *i*, $$\mu _{f\in \mathbb {F}\rightarrow i\in \mathbb {V}}$$ in Eq.  (4), requires marginalizing for the variable $$d_i$$ from the product form of joint distribution of disparity labels. The joint distribution is comprised of product of a potential function with $$(Q-1)$$ variable-to-factor messages. Factor node message related to $$d_i$$ is obtained by marginalizing a maximum of $$(Q-1)$$ neighboring variables $$\mathbb {D}_f\setminus d_i$$. For each variable being marginalized, there are *Q* product operations i.e. ($$Q - 1) + 1$$. Because there are at most *D* disparity levels, marginalizing each variable requires *D* summation terms. Therefore, there are $$(D^{Q-1} \, Q)$$ computations for each level of the disparity variable $$d_i$$. With *D* levels of $$d_i$$, there are $$D^Q\, Q$$ calculations for preparing a dependency factor-to-variable node message. In contrast, no calculations are required for preparing a message for sending from an evidence factor node to its corresponding variable node. Therefore, the FGS algorithm has a polynomial time complexity of $$O\left( MN\, D^Q\, Q\right)$$ per iteration including the computations required for preparing messages from each of the *MN* dependency factor nodes. It can be observed that far fewer number of calculations are required for preparing a message for sending from a variable node $$\mu _{f\in \mathbb {F}\rightarrow i\in \mathbb {V}}$$ in Eq.  (4) with a complexity of $$O\left( MN\, D\, (Q-1)\right)$$ and therefore does not affect the overall complexity of the FGS algorithm.

As described earlier, the following key features were employed to further reduce the computational requirements of the FGS algorithm.

1. $(D^Q\, Q)$ number of computations are eliminated when the state of the *i*th variable node $d_i$ is different from those of its neighboring nodes by using a binary potential function $\psi _f\left( \mathbb {D}_f\right)$ in the factor node-to-variable node messages $\mu _{f\in \mathbb {F}\rightarrow i\in \mathbb {V}}$ in Eq.  (4). Interaction among neighboring nodes can be further increased with appropriate changes to the potential function.
2. The number of disparity levels *D* in each of the $(Q-1)$ marginalization operations required for preparing factor node messages are significantly lowered by using a sparse cost volume for estimating a priori disparity in Eq. (9).
3. The number of factor node messages were reduced by connecting only highly correlated or dependent variable nodes (with a high bilateral filter coefficient cutoff) to each of the spatial dependency factor nodes.

### Performance metrics

For performance evaluation, we used the common performance metrics available for assessing the accuracy of the estimated disparity maps namely, the disparity error maps, peak signal-to-noise ratio (PSNR), and average absolute error (Avg. err in pixels). Disparity error maps were computed as location-wise difference between the estimated disparity $\hat{d}(x,y)$ and its ground-truth *d*(*x*, *y*) as $\hat{d}(x,y) - d(x,y)$. PSNR provides a measure of similarity between an estimated disparity map $\hat{d}(x,y)$ of size $M \times N$ pixels and the ground-truth disparity map *d*(*x*, *y*) as follows.

12 $$\begin{aligned} PSNR=10 \log _{10}\frac{255^2 \times M \times N}{\sum _{\forall (x,y)}\left( \hat{d}(x,y)-d(x,y)\right) ^2} \end{aligned}$$

A thresholded average disparity error metric with a disparity threshold of *T* pixels was defined as

13 $$\begin{aligned} Bad=\left( \frac{1}{MN}\sum _{\forall (x,y)}(|\hat{d}(x,y)- d(x,y)|>T)\right) \times 100 \end{aligned}$$

Average disparity errors were assessed at two disparity threshold levels of $T=2$ pixels (Bad2.0) and $T=0$ pixels (Avg. err).

In the majority of the stereo pairs tested, the FGS algorithm converged between 25 and 30 iterations based on the convergence measure in Eq. (8).

### Filter selection for identifying FGS variable node neighbors

**Figure 4:**
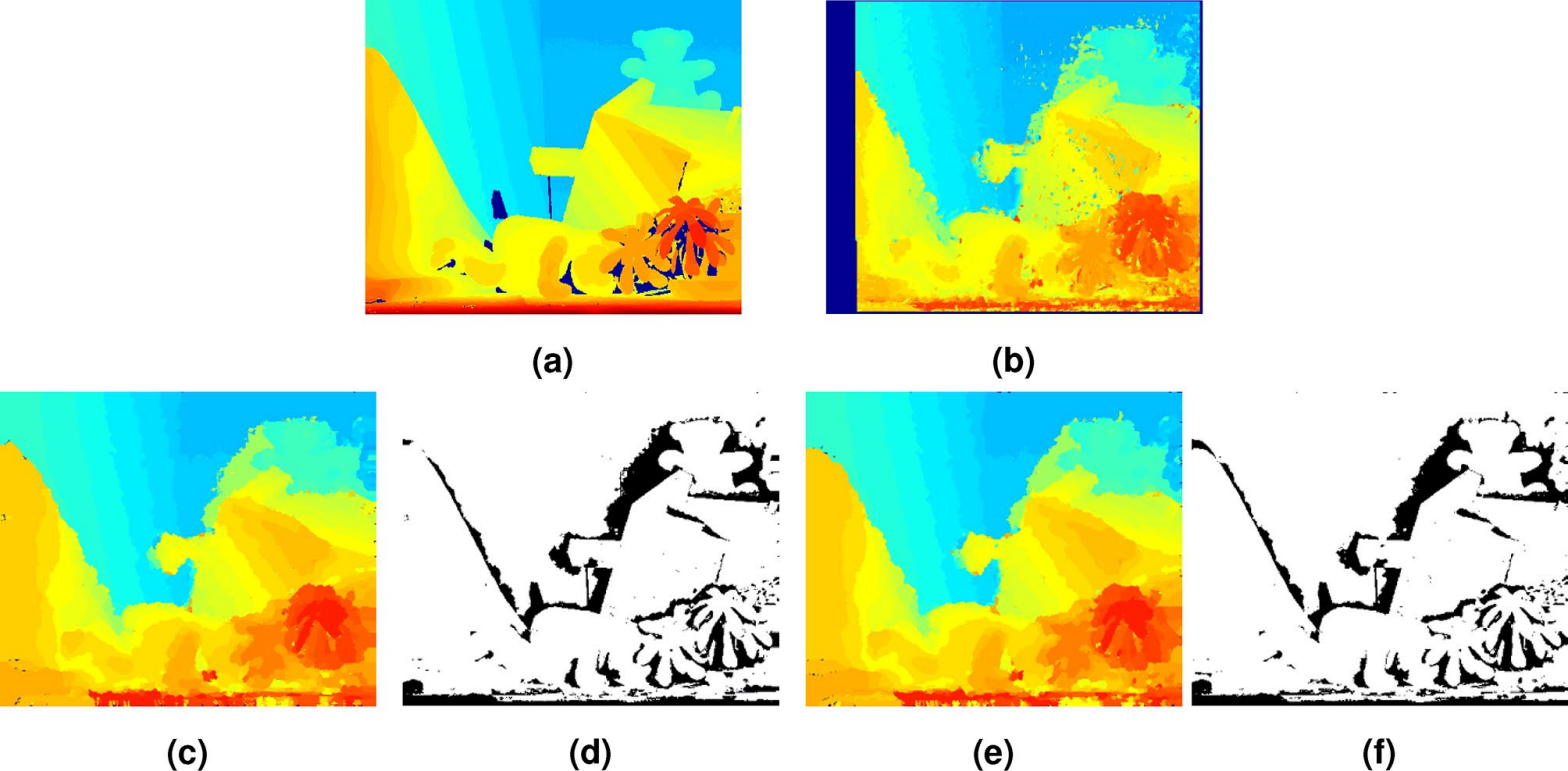
Effect of the edge-preserving filters on the accuracy of the disparity map for the “Teddy” stereo pair estimated using the FGS algorithm. Disparity estimates in the occluded regions are not excluded in the final disparity map. (**a**) Ground truth, (**b**) Initial disparity map, (**c**) Disparity map estimated using the FGS algorithm with guided filters, (**d**) Disparity error map for Fig. 4c, (**e**) Disparity map estimated using the FGS algorithm with bilateral filters, and (f) Disparity error map for Fig. 4e.

The edge-preserving filters (Sect. ‘Determining variable nodes associated with each dependency factor node’) with the highest accuracy and computational speed were selected for identifying neighboring variable nodes for each of the dependency factor nodes in the FGS algorithm. Figure 4c and e show the disparity maps for the “Teddy” stereo pair (ground truth disparity shown in Fig. 4a) estimated using guided image filters and bilateral filters respectively based on an initial disparity shown in Fig. 4b. Disparity error maps for the guided image filter and bilateral filter are shown in Fig. 4d and f respectively.

Quantitative performance measures of the edge-preserving filters are presented in Table 2. The accuracy of the FGS algorithm with guided filters (based on the Avg. err, PSNR, and Bad2.0 metrics) were slightly better than the bilateral filters. Because the guided filters resulted in a larger number of neighboring variable nodes, the run time of the FGS algorithm, however, was higher with the guided filters when compared to the FGS algorithm with bilateral filters. Therefore, for further evaluation of the FGS algorithm, bilateral filtering was chosen as the optimal choice for identifying neighboring variable nodes in the FGS factor graph.

**Table 2:** Performance of the edge-preserving filters used for identifying variables nodes neighboring each dependency factor node in the FGS factor graph.

Algorithm	Avg. err	PSNR (dB)	Bad 2.0 (%)
Initial disparity using HCS	5.18	26.33	24.21
FGS with guided filtering	**2.59**	**32.04**	**14.12**
FGS with bilateral filtering	2.60	32.02	14.17

Significant values are in bold.

### Detailed assessment of the FGS algorithm using selected stereo pairs

For detailed quantitative and qualitative assessment of the FGS algorithm, we utilized stereo image pairs with differing textures, illuminations, and exposure characteristics from the Middlebury stereo dataset including 2003^37^, 2005^38^, 2006^39^, and 2014^40^ stereo datasets. The stereo pairs selected for assessment were the *Teddy and Cones* stereo pair (2003), *Dolls stereo pair* (2005), *Rocks1* stereo pair (2006), and the *Motorcycle* stereo pair (2014). Assessment results based on the estimated disparity maps with and without post-processing are presented in the following sections.

#### Assessment results without post-processing

**Figure 5:**
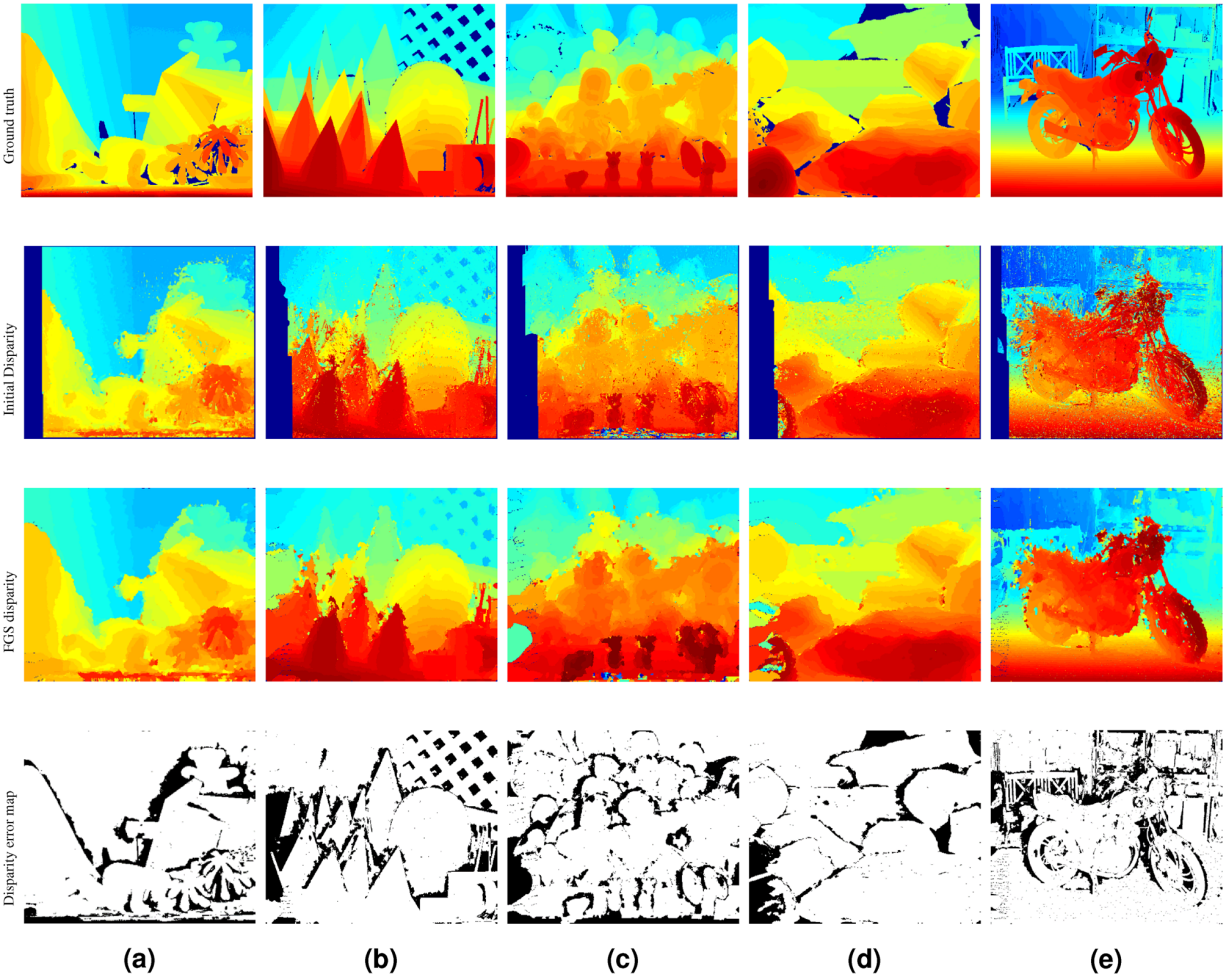
Estimated disparity maps and error maps for selected Middlebury stereo pairs without post-processing. (**a**) Teddy (dataset 2003), (**b**) Cones (dataset 2003), (**c**) Dolls (dataset 2005), (**d**) Rocks1 (dataset 2006), and (**e**) Motorcycle (dataset 2014). Disparity estimates in the occluded regions were not excluded in the final disparity maps.

For the selected stereo pairs of scenes with differing textures and scene illumination, Fig. 5 shows disparity maps estimated by the HCS algorithm without cost aggregation and post-processing, disparity maps estimated by the FGS algorithm without any post-processing, and errors in the disparity maps estimated by the FGS algorithm. A summary of the assessment metrics without post-processing the disparity maps is presented in Table 3.

Without post-processing, the FGS algorithm provided significantly lower average error (Avg. err) and percentage of bad pixels (Bad2.0) when compared to the initial disparity estimates. Higher PSNR values indicate a higher degree of similarity between the FGS estimated disparity maps and the ground truth disparity maps. Visual inspection of the FGS disparity maps in Fig. 5 revealed that the FGS algorithm is able to estimate more accurate disparities in occluded regions and generate disparity maps with well-defined boundaries. These observations validate our choice of using non-symmetric / irregular, variable and higher-order neighborhood dependency relationship among neighboring pixel locations in the FGS algorithm.

**Table 3:** Performance measures of the FGS algorithm for selected Middlebury stereo pairs without post-processing the disparity maps. Disparity estimates in the occluded regions were not excluded in the final disparity maps. FGS estimates significantly improved over the initial disparity estimates by all performance metrics.

Images	Initial			FGS Algorithm		
	Avg. err	PSNR (dB)	Bad 2.0 (%)	Avg.err	PSNR (dB)	Bad 2.0 (%)
Teddy	5.18	26.33	24.21	2.60	32.02	14.17
Cones	5.88	25.20	25.69	2.78	32.35	17.60
Dolls	7.51	23.51	29.90	3.20	30.75	22.47
Rocks1	6.35	24.42	21.97	3.29	30.15	13.58
Motorcycle	5.96	26.07	33.42	3.81	29.45	20.04

#### Assessment results with post-processing

**Figure 6:**
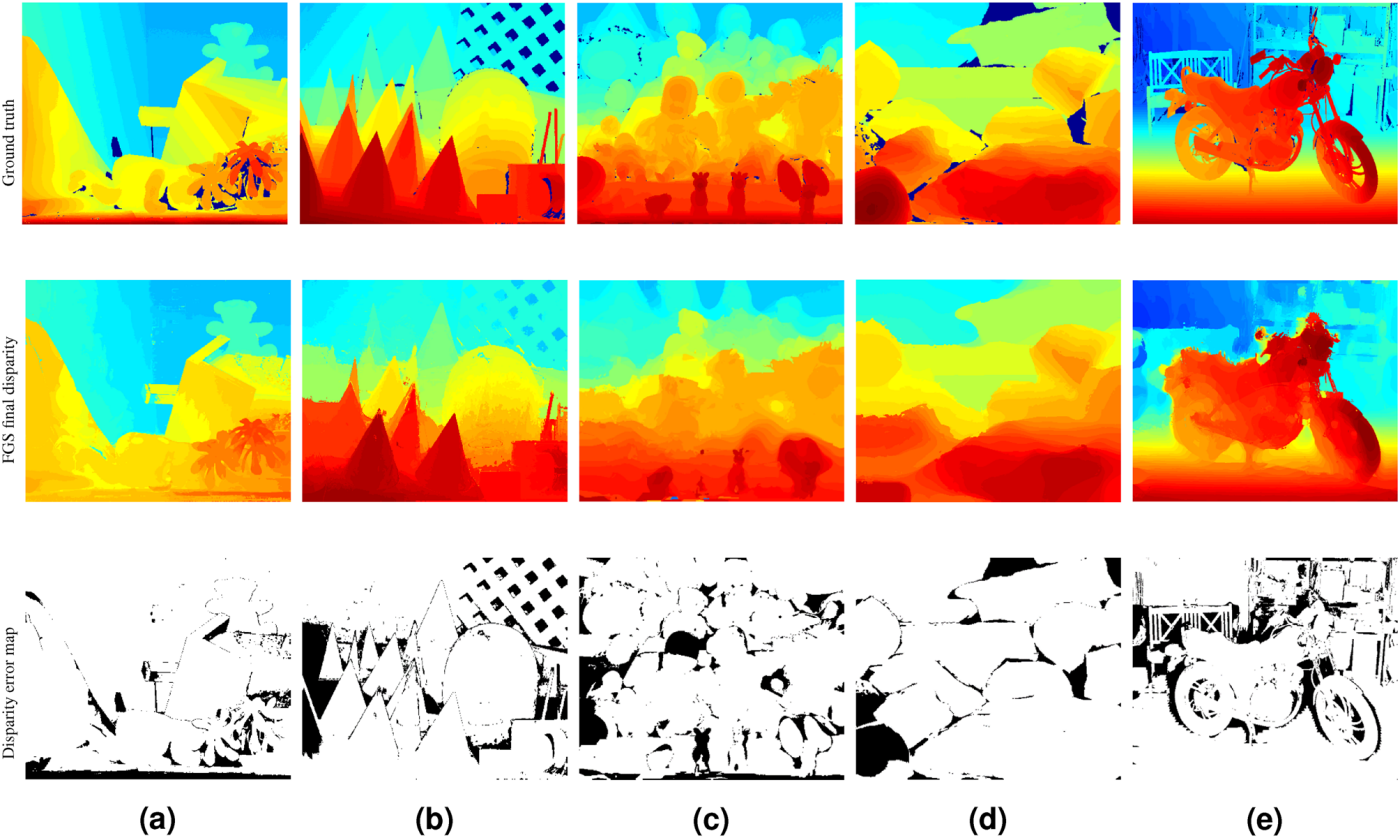
Disparity maps after post-processing and corresponding error maps for selected Middlebury stereo pairs. (**a**) Teddy (dataset 2003), (**b**) Cones (dataset 2003), (**c**) Dolls (dataset 2005), (**d**) Rocks1 (dataset 2006), and (**e**) Motorcycle (dataset 2014). Disparity estimates in the occluded regions were not excluded in the final disparity maps.

For selected stereo pairs with varying texture and illumination characteristics, Fig. 6 shows the disparity maps generated after post-processing the *maximum a posteriori* disparity estimates (shown in Fig. 5) and their corresponding disparity error maps. Table 4 presents a summary of assessment metrics after post-processing the disparity estimates. After post-processing, inconsistencies in stereo disparity estimates were corrected and disparity maps with sharper depth boundaries were generated. The average error (Avg. err) and percentage of bad pixels (Bad2.0) further decreased and the PSNR value increased after post-processing indicating higher similarity with the ground truth disparity estimates. Though the FGS disparity estimates without post-processing demonstrated higher accuracy in occluded and homogeneous regions, post-processing using a weighted median filter further reduced inconsistent disparity estimates and improved the overall quality of the disparity maps.

**Table 4:** Performance measures of the FGS algorithm for selected Middlebury stereo pairs after post-processing.

Images	FGS-based			Final		
	Avg.err	PSNR (dB)	Bad2.0 (%)	Avg.err	PSNR (dB)	Bad2.0 (%)
Teddy	2.60	32.02	14.17	1.90	33.25	9.55
Cones	2.78	32.35	17.60	2.32	33.33	15.11
Dolls	3.20	30.75	22.47	2.04	32.52	18.98
Rocks1	3.29	30.15	13.58	2.78	31.10	12.06
Motorcycle	3.81	29.45	20.04	3.36	30.02	18.87

Disparity estimates in the occluded regions were not excluded. FGS estimates significantly improved over the initial disparity estimates by all performance metrics.

### Performance of the FGS algorithm vs state-of-the-art algorithms

To the best of our knowledge, the proposed FGS algorithm is the first disparity estimation technique based on factor graphs. The performance of the non-learning-based FGS algorithm was compared with recent state-of-the-art learning-based and non-learning-based disparity estimation algorithms. All disparity estimation algorithms were evaluated using stereo pairs from the current Middlebury evaluation dataset version 3.0. In addition to the performance metrics of *Avg. err*, *PSNR* and *Bad 2.0 %*, a weighted average measure was calculated for each performance metric based on the level of difficulty of estimating the disparity map for each stereo pair in the Middlebury evaluation dataset version 3.0. A summary of all the assessment metrics and a weighted performance measure for the FGS algorithm is presented in Table 5.

**Table 5:** All performance measures of the proposed FGS algorithm for the stereo pairs in the current Middlebury evaluation dataset version 3.0.

Images	FGS-based		
(Weight)	Avg.err	PSNR (dB)	Bad2.0 (%)
Adiron (8)	03.63	30.10	17.96
ArtL (8)	04.81	30.70	44.81
Jadepl (8)	26.72	18.33	47.01
Motor (8)	03.36	30.02	18.87
MotorE (8)	05.90	27.11	28.48
Piano (8)	04.23	30.30	24.12
PianoL (4)	07.10	27.11	35.21
Pipes (8)	07.71	25.71	39.25
Playrm (4)	05.30	27.10	30.11
Playt (4)	03.19	31.98	28.10
PlaytP (8)	03.05	31.95	24.36
Recyc (8)	03.71	31.59	32.54
Shelvs (4)	05.30	27.81	34.07
Teddy (8)	01.90	33.25	09.55
Vintage (4)	10.22	29.11	54.10
**Weighted**			
**Average**	**6.45**	**28.85**	**30.22**

Significant values are in bold.

Disparity estimates in the occluded regions were not excluded in the final disparity maps.

### FGS vs non-learning based disparity estimation methods

Performance of the FGS algorithm was compared with 12 recently developed non-learning-based disparity estimation procedures including 7 local methods, 3 global methods, and one fusion method using both local and global approaches for disparity estimation. The local methods were: weighted adaptive cross-region-based guided image filtering method (ACR-GIF-OW)^54^, real-time stereo matching algorithm with FPGA architecture (MANE)^55^, adaptive support-weight approach in pyramid structure (DAWA-F)^56^, encoding-based approaches PPEP-GF^57^, absolute difference (AD) and census transform-based stereo matching with guided image filtering (ADSR-GIF)^58^, the sum of absolute difference (SAD) based stereo matching aggregated with adaptive weighted bilateral filter (SM-AWP)^59^, statistical *maximum a posteriori* estimation of MRF disparity labels (SRM)^60^. The global disparity estimation procedures chosen for comparison were: binocular narrow-baseline stereo matching procedure using a max-tree data structure (MTS)^61^ and its improvement (MTS-2)^62^, and an accelerated multi-block matching (MBM) algorithm on GPU^22^. FASW approach^63^ uses both local and global strategies and is based on a census transform with adaptive support weight.

**Table 6:** Performance (Avg. err) of the FGS algorithm vs state-of-the-art *non-learning-based disparity estimation algorithms* for stereo pairs in the Middlebury evaluation dataset version 3.0.

Images (Weight)	FGS	HCS^49^	ACR-GIF-OW^54^	MANE^55^	SRM^60^	MTS^61^	DAWA-F^56^	PPEP-GF^57^	MTS-2^62^	ADSR-GIF^58^	FASW^63^	SM-AWP^59^	MBM^22^
Adiron (8)	3.63	3.98	4.53	11.60	2.88	19.00	4.37	8.12	21.50	6.40	**2.86**	10.5	4.39
ArtL (8)	4.81	**4.31**	8.41	22.90	5.96	22.50	13.00	14.80	22.40	9.00	8.03	19.9	8.80
Jadepl (8)	26.72	27.22	**22.10**	45.90	24.70	123.00	44.40	46.90	108.00	26.10	34.70	62.7	37.60
Motor (8)	**3.36**	3.91	7.93	12.40	4.46	17.50	7.29	7.99	15.30	8.11	5.44	11:00	5.76
MotorE (8)	5.90	6.12	7.88	12.30	**4.43**	20.70	7.04	7.62	30.60	11.40	4.43	12.5	5.56
Piano (8)	4.23	5.23	6.36	15.10	5.73	13.00	**3.27**	9.76	10.00	6.15	5.54	9.08	6.67
PianoL (4)	**7.10**	7.44	27.70	24.70	7.99	32.00	21.70	18.80	26.20	34.00	10.80	29.7	12.40
Pipes (8)	7.71	7.52	11.00	22.30	**6.96**	29.40	15.90	17.30	24.60	14.90	10.80	21.11	11.80
Playrm (4)	**5.30**	6.16	8.51	31.10	10.70	26.90	8.86	19.30	23.30	10.50	7.31	20.7	12.90
Playt (4)	**3.19**	3.57	16.10	39.90	4.48	27.40	6.39	45.50	12.70	16.70	14.50	9.5	12.00
PlaytP (8)	**3.05**	3.17	6.60	17.30	3.32	12.00	3.34	24.50	9.29	10.00	3.32	9.75	6.37
Recyc (8)	3.71	3.62	4.26	9.67	2.92	17.50	3.89	7.64	11.00	4.20	**2.84**	7.18	3.67
Shelvs (4)	**5.30**	5.51	13.10	22.50	7.41	12.10	11.10	17.20	11.80	9.97	8.70	11.4	11.80
Teddy (8)	**1.90**	2.10	2.86	12.50	1.92	8.11	3.39	7.11	6.67	3.35	2.83	9.44	3.74
Vintage (4)	10.22	10.62	7.77	51.00	15.80	27.20	**6.48**	23.40	33.80	10.90	6.79	16.8	14.10
**Weighted**													
**Average**	**6.45**	6.71	9.48	21.33	6.92	27.64	10.65	17.11	25.06	11.25	8.39	17.38	10.08

The weight of a stereo pair represents the level of difficulty in estimating disparity map from the respective stereo pair. The algorithm with the highest performance is highlighted in bold.

Avg. err metric and a weighted average of Avg. err for the FGS algorithm and the non-learning-based disparity estimation procedures are presented in Table 6. Image weight given in Table 6 represents the level of difficulty in estimating the disparity from a given stereo pair. In general, the proposed FGS algorithm provided higher accuracy for all the stereo pairs comparable to that of other non-learning-based methods. The FGS method provided the lowest weighted average of Avg. err of 6.45 pixels among all the non-learning-based methods. Further, the FGS method provided the lowest estimation error (Avg. err) for 3 out of the 10 difficult stereo pairs (image weight = 8) and for 4 out of the 5 moderately difficult stereo pairs (image weight = 4). Among the remaining 7 difficult stereo pairs, HCS method provided lower error for 1 pair, ACR-GIF-OW method for 1 pair, SRM method for 2 pairs, DAWA-F method for 1 pair, and FASW for 2 pairs. The DAWA-F method provided the lowest estimation error for the remaining 1 moderately difficult stereo pairs.

### FGS vs learning-based disparity estimation methods

Performance of the FGS algorithm was also compared with the following recently developed learning-based disparity procedures: a method based on a fusion of convolutional neural networks (CNN) and conditional random fields (LBPS)^64^, a fully convolutional-densely connected neural network (FC-DCNN)^27^, a deep-learning assisted method to produce initial estimate with Semi-Global Block Matching method (SGBMP)^65^, a deep learning-based self-guided cost aggregation method (DSGCA)^66^, a stereo matching algorithm with a pretrained network and global energy minimization SIGMRF^67^, a multi-dimensional convolutional neural network (MSMD-ROB)^68^ and a CNN-based network using ResNet (CBMBNet)^69^.

**Table 7:** Performance (Avg. err) of the FGS algorithm vs state-of-the-art *learning-based disparity estimation algorithms* for stereo pairs in the Middlebury evaluation dataset version 3.0.

Images (Weight)	FGS	FC-DCNN^27^	LBPS^64^	SGBMP^65^	DSGCA^66^	SIGMRF^67^	MSMD-ROB^68^	CBMBNet^69^
Adiron (8)	3.63	2.87	1.92	6.50	7.68	3.07	2.85	**1.63**
ArtL (8)	**4.81**	6.30	7.02	9.33	21.70	7.83	8.58	8.89
Jadepl (8)	26.72	32.70	**24.9**	56.80	45.00	32.80	45.10	27.70
Motor (8)	**3.36**	4.65	4.12	4.04	10.60	5.83	5.12	4.19
MotorE (8)	5.90	4.58	**4.09**	5.43	10.40	5.92	4.99	4.12
Piano (8)	4.23	4.45	**3.02**	4.77	11.50	5.38	3.75	3.22
PianoL (4)	7.10	9.25	**3.63**	14.80	24.50	8.13	7.18	5.40
Pipes (8)	7.71	10.00	**7.37**	7.85	19.90	11.30	11.00	8.03
Playrm (4)	5.30	6.15	**4.83**	7.62	24.60	5.66	6.86	5.96
Playt (4)	**3.19**	9.60	3.20	10.60	34.50	13.40	9.74	5.69
PlaytP (8)	**3.05**	3.26	3.39	3.78	14.80	4.26	9.32	3.89
Recyc (8)	3.71	2.67	1.71	3.19	7.56	3.07	2.74	**1.7**
Shelvs (4)	5.30	10.00	**3.19**	5.00	17.30	8.57	3.56	7.70
Teddy (8)	**1.9**	2.17	2.33	3.35	12.20	2.76	3.02	4.55
Vintage (4)	10.22	9.34	**3.18**	30.00	43.80	15.50	9.59	5.71
**Weighted**								
**Average**	6.45	7.67	**5.51**	10.38	18.70	8.63	9.19	6.65

The weight of a stereo pair represents the level of difficulty in estimating disparity map from the respective stereo pair. The algorithm with the highest performance is highlighted in bold.

Avg. err metric for the FGS algorithm and the learning-based disparity estimation procedures are presented in Table 7. Among the recent learning-based disparity estimation procedures, the LBPS method provided the least weighted Avg. err of 5.51 for all stereo pairs, provided the least Avg. error for 4 out of 10 difficult stereo pairs and the least Avg. err for 4 out of 5 moderately difficult stereo pairs. The FGS method provided the second-lowest weighted average of Avg. err of 6.45 for all stereo pairs, provided the least Avg. err for 4 out of 10 difficult stereo pairs and the least Avg. error for 1 out of 5 moderately difficult stereo pairs. The CBMBNet provided the third lowest weighted Avg. err of 6.65 and the least Avg. err for 2 out of 10 difficult stereo pairs.

## Conclusions

We have presented a new probabilistic factor-graph-based disparity estimation algorithm that improves the accuracy of disparity estimates in stereo image pairs with varying texture and illumination characteristics by enforcing spatial dependencies among scene characteristics as well as among disparity estimates. In contrast to MRF models, our factor graph formulation allows a larger as well as a spatially variable neighborhood system dependent only on the local scene characteristics. Our factor graph formulation can be used for obtaining *maximum a posteriori* estimates from models or optimization problems with complex dependency structure among hidden variables. The strategies of using a priori distributions with shorter support and spatial dependencies are useful for improving the computational speed of message convergence in factor graph-based inference problems. We rigorously evaluated the performance of the new factor-graph-based disparity estimation algorithm using Middlebury benchmark stereo datasets^37–39^, and^40^. Our experimental results indicate that the factor-graph algorithm provides disparity estimates with higher accuracy when compared to recent non-learning as well as learning-based disparity estimation algorithms using Middlebury evaluation dataset version 3.0^5^. The factor-graph algorithm may also be useful for other dense estimation problems such as optical flow estimation.

## Data Availability

Stereo datasets that were used to evaluate the proposed methods are publicly available from the following URLS: (1) https://vision.middlebury.edu/stereo/data/scenes2003/. (2) https://vision.middlebury.edu/stereo/data/scenes2005/. (3) https://vision.middlebury.edu/stereo/data/scenes2006/. (4) https://vision.middlebury.edu/stereo/data/scenes2014/.
